# Supplementary material for: Halide Perovskite Inducing Anomalous Nonvolatile Polarization in Poly(vinylidene fluoride)-based Flexible Nanocomposites
Source: Nat Commun. 2024 May 10;15:3943. doi: 10.1038/s41467-024-48348-4 (PMC11087492; doi:10.1038/s41467-024-48348-4)
Supplement: Supplementary file 1 — Supplementary Information [file 41467_2024_48348_MOESM1_ESM.pdf]

Supplementary Information

Halide Perovskite Inducing Anomalous Nonvolatile Polarization in Poly(vinylidene fluoride)-based Flexible Nanocomposites

Yao Wang[1*], Chen Huang[1], Ziwei Cheng[1], Zhenghao Liu[2,3], Yuan Zhang[2,3], Yantao Zheng[1], Shulin Chen[4], Jie Wang[5], Peng Gao[4,6], Yang Shen[7], Chungang Duan[8], Yuan Deng[9], Ce-Wen Nan[7], Jiangyu Li[2,3*]

[1] School of Materials Science and Engineering, Beihang University, Beijing 100191, China

[2] Department of Materials Science and Engineering, Southern University of Science and Technology, Shenzhen 518055, Guangdong, China

[3] Guangdong Provincial Key Laboratory of Functional Oxide Materials and Devices, Southern University of Science and Technology, Shenzhen 518055, Guangdong, China

[4] International Center for Quantum Materials and Electron Microscopy Laboratory, School of Physics, Peking University, Beijing 100871, China

[5] Department of Engineering Mechanics, Key Laboratory of Soft Machines and Smart Devices of Zhejiang Province, Zhejiang University, Hangzhou 310027, Zhejiang, China

[6] Collaborative Innovation Center of Quantum Matter, Beijing 100871, China

[7] School of Materials Science and Engineering, State Key Lab of New Ceramics and Fine Processing, Tsinghua University, Beijing 100084, China

[8] State Key Laboratory of Precision Spectroscopy and Key Laboratory of Polar Materials and Devices, Ministry of Education, Department of Electronics, East China Normal University, Shanghai 200241, China

[9] Key Laboratory of Intelligent Sensing Materials and Chip Integration Technology of Zhejiang Province, Hangzhou Innovation Institute, Beihang University, Hangzhou 310052, Zhejiang, China.

*Corresponding author. E-mail: wang-yao@buaa.edu.cn (Y.W.); lijy@sustech.edu.cn (J.L.)

Supplementary Note 1. Formation of nanocomposite film

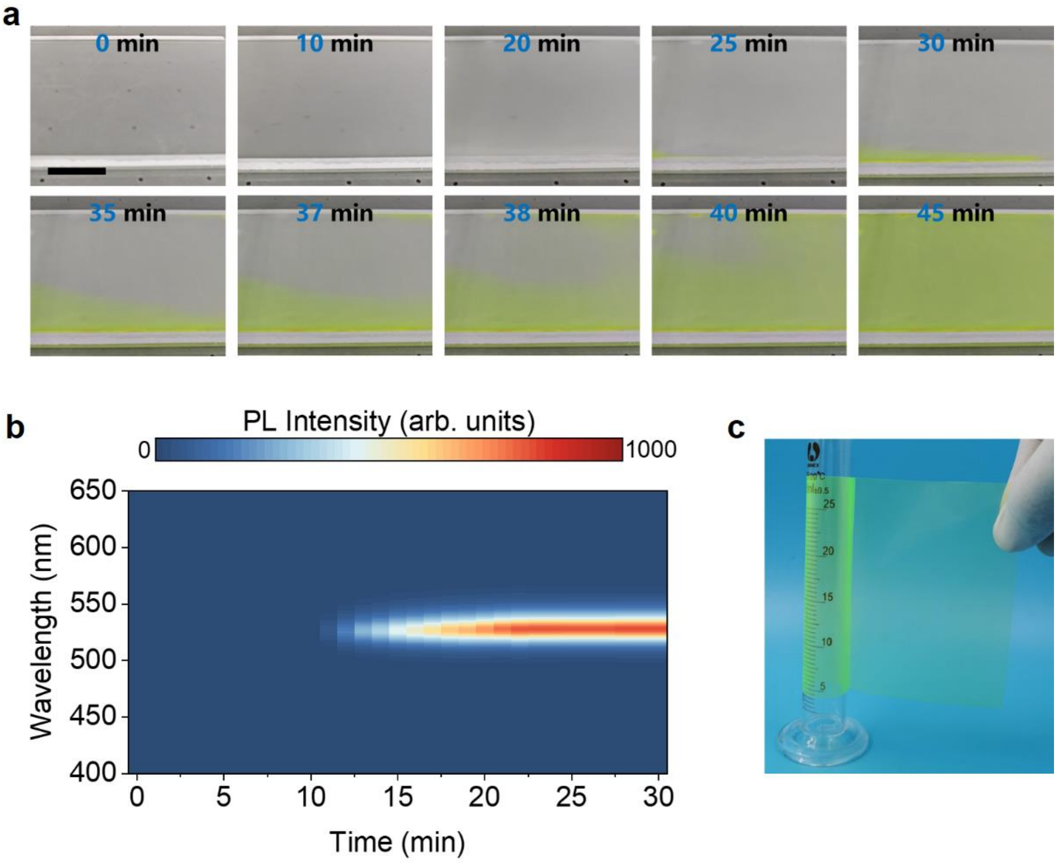

Supplementary Fig. 1 Formation of MAPB/PVDF nanocomposite film. a Pictures recorded from the film formation process showing nucleation of MAPB nanocrystals and PVDF film. The scale bar is 5 cm. **b** in situ PL spectra of MAPB/PVDF nanocomposite film during film formation process. **c** Photo of a highly homogenous piece of nanocomposite film rolled around a cylinder.

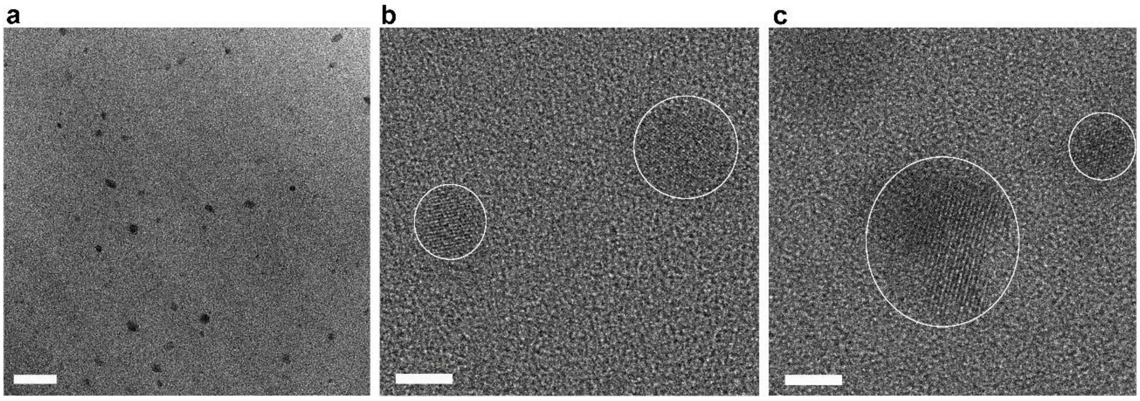

Supplementary Fig. 2 Microstructure of MAPB/PVDF nanocomposite film. a TEM image of MAPB/PVDF film. The scale bar is 50 nm. **b, c** HRTEM images of two well separated MAPB nanocrystals taken from different regions. The scale bar is 5 nm.

Supplementary Note 2. Crystallization behavior

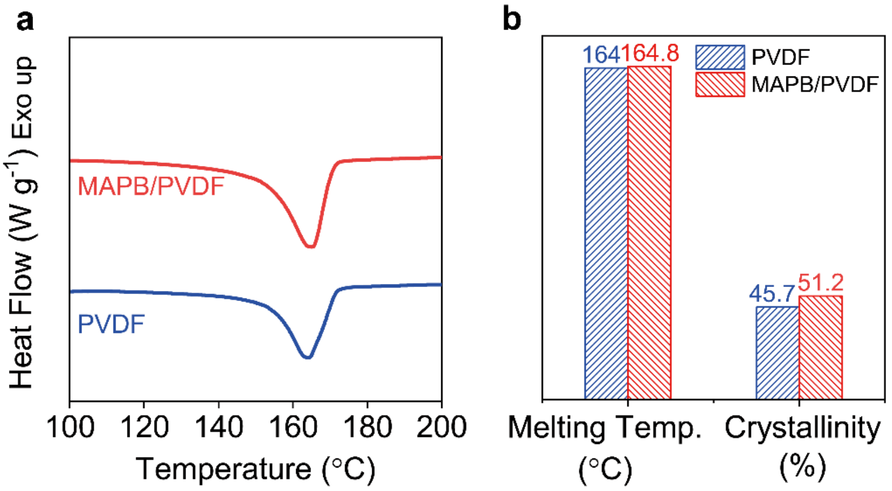

Supplementary Fig. 3 Crystallization behavior of PVDF and MAPB/PVDF nanocomposite films. a DSC curves. **b** Calculated crystallinity and melting temperature of the films.

Supplementary Note 3. Phase analysis

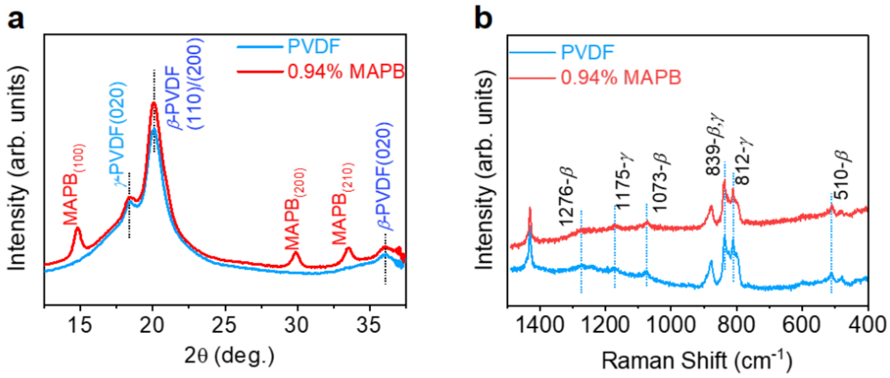

Supplementary Fig. 4 Crystalline phase analysis of PVDF and MAPB/PVDF nanocomposite films. a 1D-WAXS patterns. **b** Raman spectra.

Supplementary Note 4. Photoluminescence property of MAPB nanoparticles

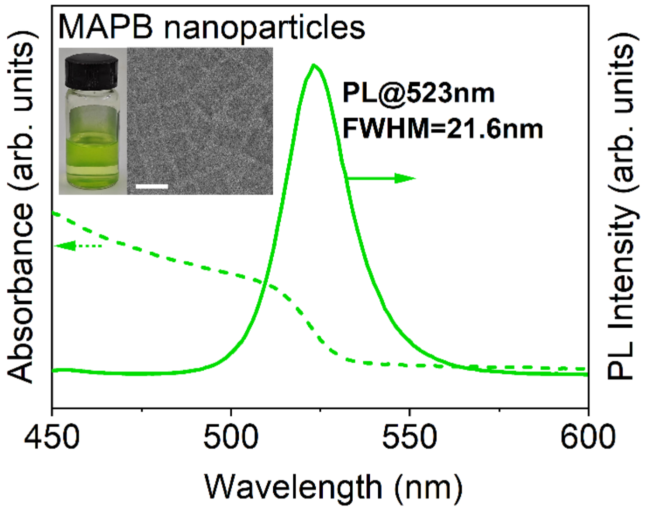

Supplementary Fig. 5 Optical properties of MAPB nanoparticles. UV-vis absorption and photoluminescence spectra of MAPB nanoparticles in n-hexane solvent with the inset showing the photo of the MAPB NPs dispersion and their morphology observed by TEM. The scale bar is 20 nm.

Supplementary Note 5. Performance Stability

5.1 Stability of the large polarization behavior

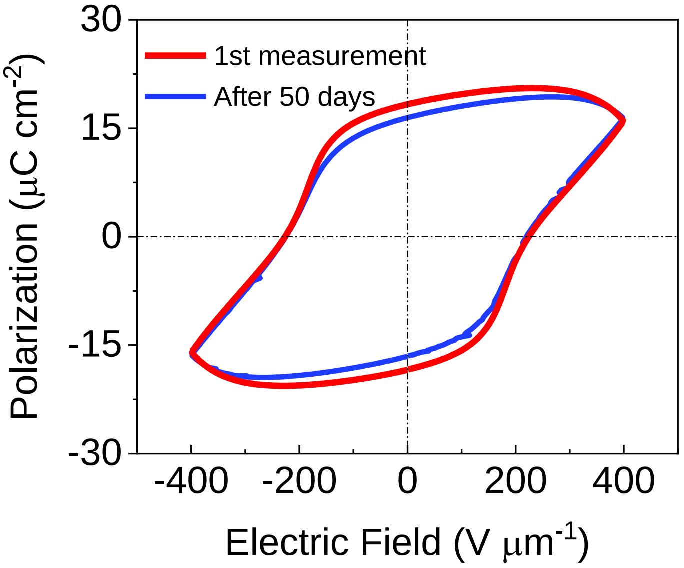

Supplementary Fig. 6 Polarization stability test of MAPB/PVDF nanocomposite films. Comparison on initial *P-E* loop of 0.94% MAPB/PVDF nanocomposite and loop measured after 50 days.

5.2 Photoluminescence stability in water

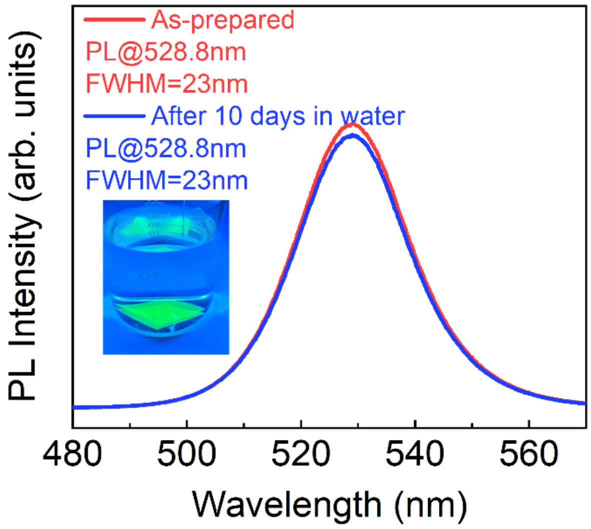

Supplementary Fig. 7 Photoluminescence stability test of MAPB/PVDF nanocomposite film. Comparison on PL spectra of MAPB/PVDF film before and after soaking in water for 10 days.

Supplementary Note 6. Dielectric properties

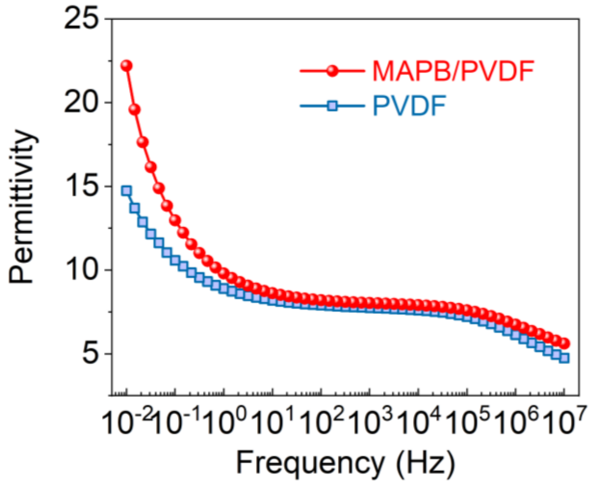

Supplementary Fig. 8 Frequency-dependent dielectric properties of PVDF and 0.94% MAPB/PVDF films. Permittivity versus frequency measured at room temperature.

Supplementary Note 7. Second harmonic generation

The SHG measurements were carried out by transmitted far-field SHG polarimetry system, as shown in Supplementary Fig. 6a. Notably, the incident angle θ is 30° instead of normal incident, since the out-of-polarization cannot be detected under normal incident in tetragonal 4*mm*, orthorhombic *mm*2 and hexagonal 6*mm* symmetries[1]. The nonlinear polarization generated from the sample can be written as $P_i^{2\omega} \propto d_{ijk} E_j E_k$, where E is the electric field of incident optical, and the d_{ijk} is nonlinear optical coefficient tensor. The electric fields have components of $E = (E_0\cos(\varphi), E_0\sin(\varphi), 0)$ in (x, y, z) lab system, with the fundamental light propagating in z-direction by rotating the polarization angle through a half-wave plate (φ = 0 ° is along x-axis). The all-trans β-phase of PVDF has symmetry of orthogonal *mm*2[2], and its nonlinear optical tensor can be written in crystal physics axes (X, Y, Z) with the Z axis along out-of-plane polarization:

$$d_{ijk} = \begin{pmatrix} 0 & 0 & 0 & 0 & d_{15} & 0 \\ 0 & 0 & 0 & d_{24} & 0 & 0 \\ d_{31} & d_{32} & d_{33} & 0 & 0 & 0 \end{pmatrix} \qquad (1)$$

The nonlinear polarization generated by the PVDF is then calculated by transforming the nonlinear tensor to the lab system with the rule $T_{ijk}^{\mathrm{new}} = a_{il} a_{jm} a_{kn} T_{lmn}^{\mathrm{old}}$, where T is the third rank property tensor, and $a_{il} = \hat{e}_i^{\mathrm{new}} \cdot \hat{e}_j^{\mathrm{old}}$ (i, l = 1, 2, 3) is the corresponding coordinate transformation matrix[3]. The calculated nonlinear optical tensor in lab system can be written as:

$$d_{ijk}^{\mathrm{new}} = \begin{pmatrix} -(\frac{3d_{15}}{4}+\frac{3d_{31}}{8}+\frac{d_{33}}{8}) & -\frac{d_{32}}{2} & (\frac{3d_{15}}{4}-\frac{d_{31}}{8}-\frac{3d_{33}}{8}) & 0 & (\frac{\sqrt{3}d_{31}}{8}-\frac{\sqrt{3}d_{14}}{4}-\frac{\sqrt{3}d_{33}}{8}) & 0 \\ 0 & 0 & 0 & -\frac{\sqrt{3}d_{24}}{2} & 0 & -\frac{d_{24}}{2} \\ (\frac{\sqrt{3}d_{15}}{4}-\frac{3\sqrt{3}d_{31}}{8}-\frac{\sqrt{3}d_{33}}{8}) & -\frac{\sqrt{3}d_{32}}{2} & -(\frac{\sqrt{3}d_{15}}{4}+\frac{\sqrt{3}d_{31}}{8}+\frac{3\sqrt{3}d_{33}}{8}) & 0 & (\frac{d_{15}}{4}+\frac{3d_{31}}{8}-\frac{3d_{33}}{8}) & 0 \end{pmatrix}$$

(2)

Therefore, the nonlinear polarization is be given by:

$$P^{2\omega} = \begin{pmatrix} P_1 \\ P_2 \\ P_3 \end{pmatrix} = d_{ijk}^{\text{new}} \begin{pmatrix} E_1^2 \\ E_2^2 \\ E_3^2 \\ 2E_2E_3 \\ 2E_1E_3 \\ 2E_1E_2 \end{pmatrix} = d_{ijk}^{\text{new}} \begin{pmatrix} E_0^2\cos(\varphi)^2 \\ E_0^2\sin(\varphi)^2 \\ 0 \\ 0 \\ 0 \\ 2E_0^2\cos(\varphi)\sin(\varphi) \end{pmatrix} \tag{3}$$

As the measured SHG intensities with *p*-output polarization is calculated as $I_{\text{p}}^{2\omega} = |P_1|^2$, the expressions for $I_{\text{p}}^{2\omega}$ becomes when $E_0 = 1$:

$$I_{\text{p}}^{2\omega} = \left(-(\frac{3d_{15}}{4} + \frac{3d_{31}}{8} + \frac{d_{33}}{8})\cos(\varphi)^2 - \frac{d_{32}}{2}\sin(\varphi)^2 \right)^2 \tag{4}$$

The measured *p*-out polarized SHG polar plots can be fitted well based on equation (4).

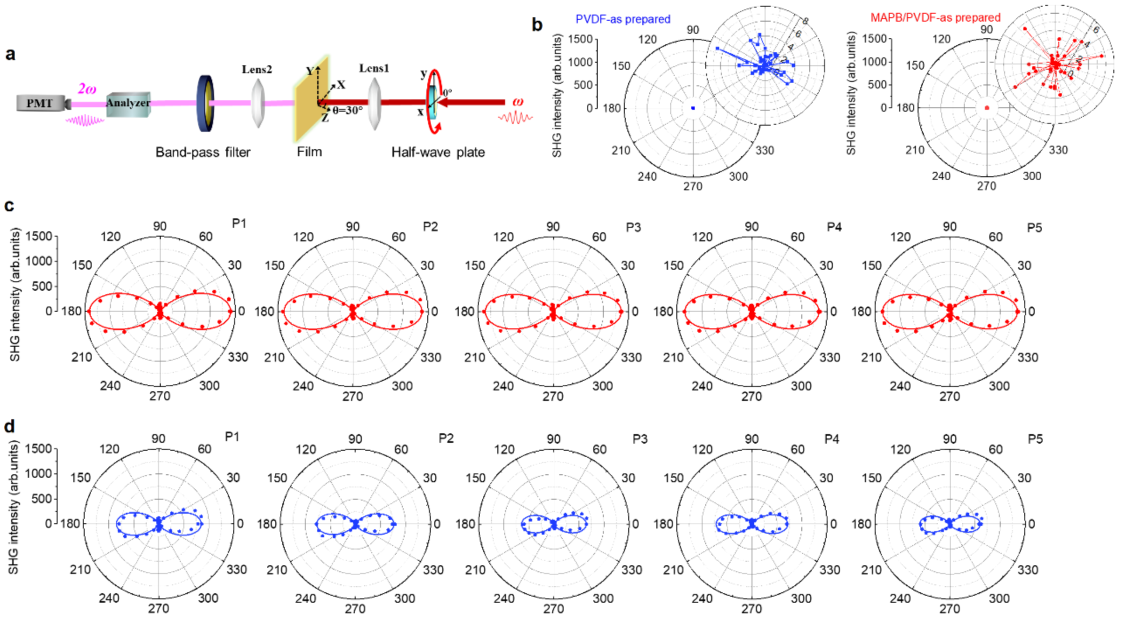

Supplementary Fig. 9 SHG measurement for as-prepared and poled PVDF and MAPB/PVDF films. a Experimental setup of the transmitted far-field SHG polarimetry system. **b** Polar plots of transmitted SHG intensity generated from as-prepared PVDF and MAPB/PVDF films with the insets showing enlarged SHG signals at very low intensity. Multiple points SHG measurement for poled films: **c** MAPB/PVDF and **d** PVDF, respectively.

Supplementary Note 8. Electrostrictive properties

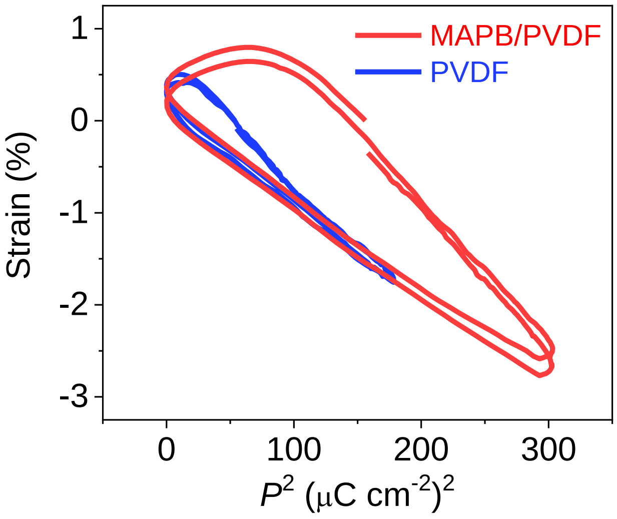

Supplementary Fig. 10 Electrostrictive properties of PVDF and MAPB/PVDF films. Strain vs. P^2 curves reveal that the electrostrictive coefficient Q_{33} of MAPB/PVDF nanocomposite film is the same as that of PVDF film, based on the relationship $S_3 = Q_{33}P^2$

Supplementary Note 9. Mechanical stability of the large polarization

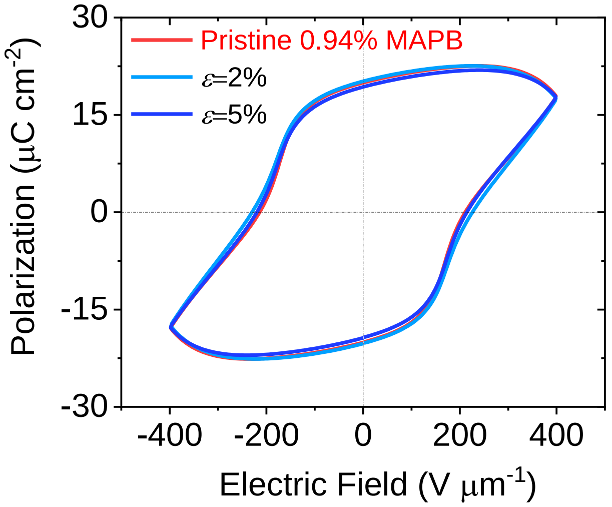

Supplementary Fig. 11 Mechanical stability test on MAPB/PVDF nanocomposite film. *P-E* hysteresis loops of MAPB/PVDF film after stretched at strain 2% and 5% in comparison with pristine film.

Supplementary Note 10. Sequential excitation scanning probe microscopy

PFM mappings were acquired via a custom developed sequential excitation technical[4,5]. The dynamics of the interaction during PFM measurement can be described well by a simple harmonic oscillator (SHO) model[6]:

$$A(\omega)=\frac{A_0\omega_0^2}{\sqrt{(\omega_0^2-\omega^2)^2+(\omega_0\omega/Q)^2}} \tag{5}$$

and

$$\tan\varphi(\omega)=\frac{\omega_0\omega}{Q(\omega_0^2-\omega^2)} \tag{6}$$

where A_0, ω_0, and Q are intrinsic electromechanical response (piezoelectricity), resonant frequency (elasticity) and quality factor (energy dissipation) of the system. In order to capture these data accurately, a series of single frequency PFM data were acquired under AC voltage of 6 V and excitation frequency ranging from 300 kHz to 400 kHz with 2 kHz increment. The range of excitation frequency is determined from a preliminary PFM scan that surveys the distribution of resonance frequency (ω_0) over scanned region, so that the adopted frequency range covers the resonance frequencies of all the points. As such, no resonance tracking is necessary. These data are then fitted by equations (5) and (6) to determine A_0, ω_0, and Q, respectively.

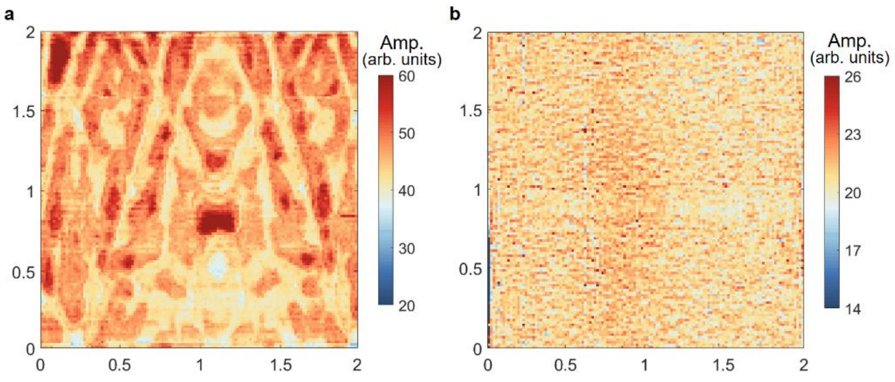

Supplementary Fig. 12 Amplitude mapping collected from second harmonic responses from the MAPB/PVDF nanocomposite films. **a** Before and **b** after poling with a scanning area of 2 μm × 2 μm.

Supplementary Note 11. Poling effects

11.1 Poling induced variation in phase structure

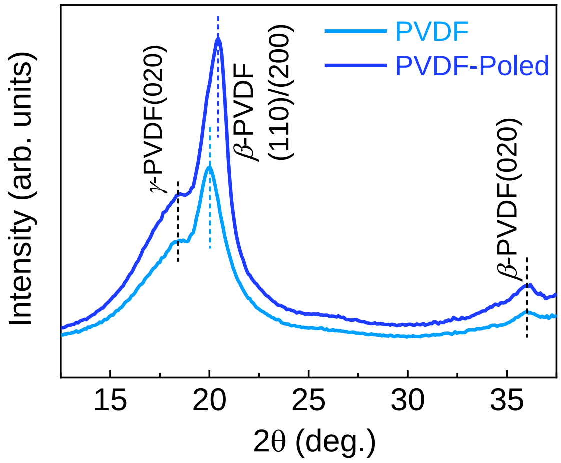

Supplementary Fig. 13 Poling induced variation in crystalline structure of PVDF film. 1D-WAXS patterns of PVDF film before and after poling.

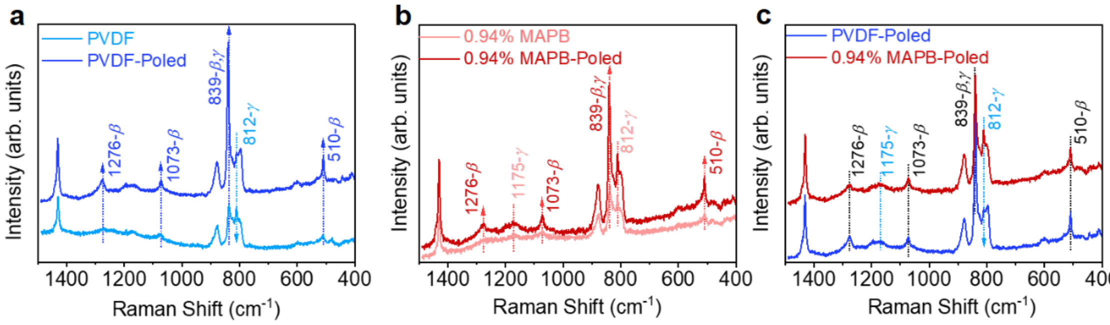

Supplementary Fig. 14 Comparison on Raman spectra of MAPB/PVDF and PVDF films before and after poling. a PVDF, **b** MAPB/PVDF and **c** poled MAPB/PVDF and PVDF.

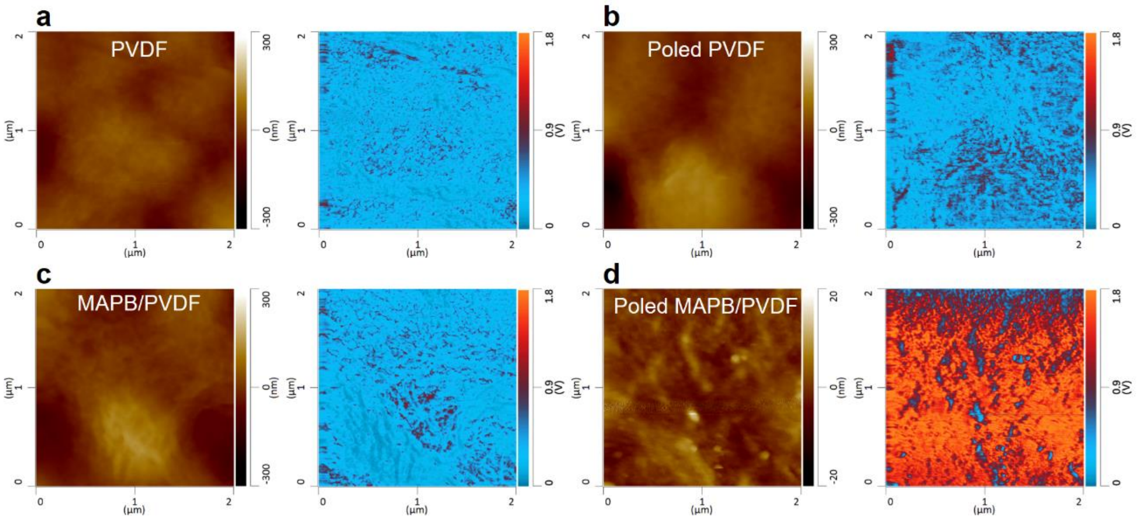

Supplementary Fig. 15 AFM-IR characterization of new IR vibrating mode arising from poled MAPB/PVDF nanocomposite. Simultaneously obtained topography (left) and AFM-IR chemical maps (right) with IR laser irradiating at 1260 cm^{-1} of **a** PVDF, **b** poled PVDF, **c** MAPB/PVDF and **d** poled MAPB/PVDF. The scanning area is 2 μm × 2 μm.

11.2 Poling induced variation in optical properties

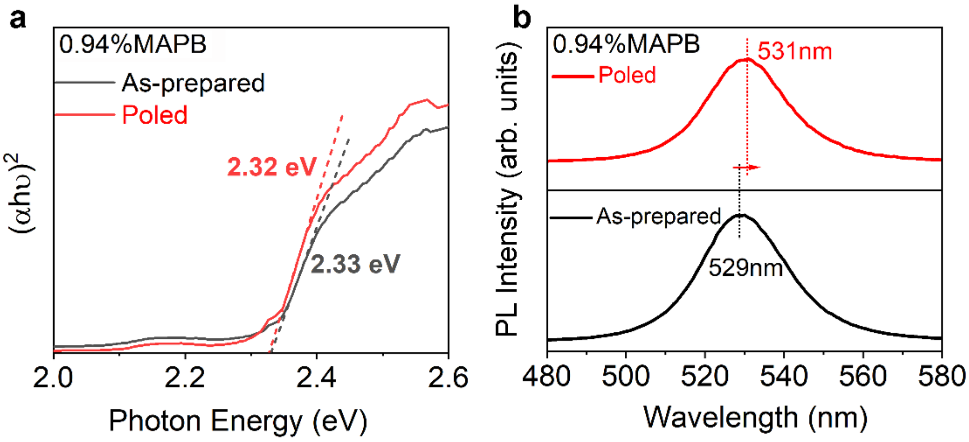

Supplementary Fig. 16 Poling induced variation in optical properties of MAPB/PVDF nanocomposite. **a** UV-vis absorption spectra, and **b** PL spectra of the MAPB/PVDF nanocomposite film before and after poling.

Supplementary Note 12. Molecular Dynamic Simulations

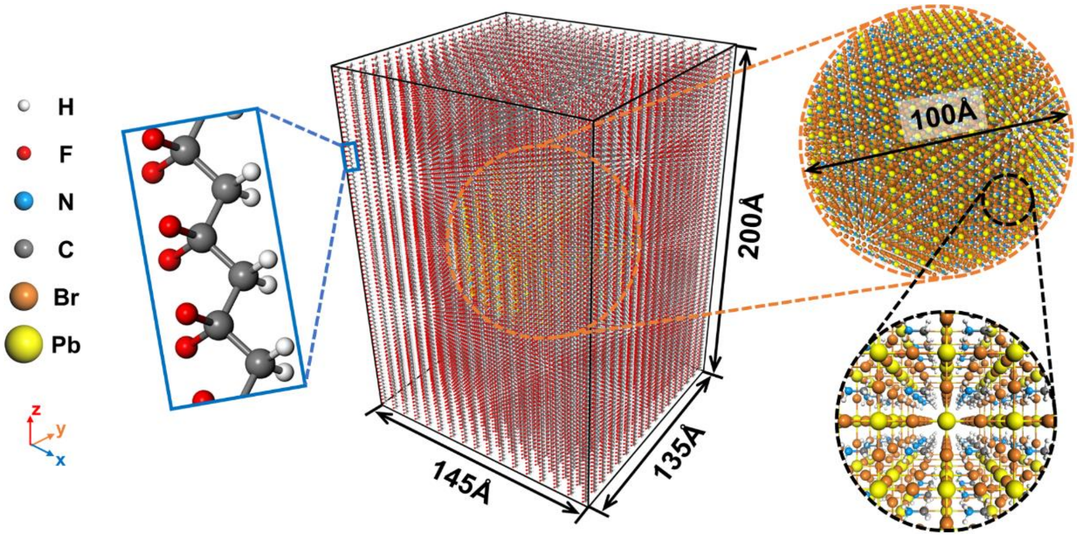

Supplementary Fig. 17 Molecular model of MAPB/PVDF nanocomposite. A single MAPB crystal with diameter of 100 Å locates inside PVDF chains arranged along z-axis and the size of simulation box is 145 Å × 135 Å × 200 Å. The detailed atomic structures of PVDF chain segment and MAPB crystal are enlarged in the left frame and right circle, respectively.

Materials Studio software 17.1 (MS, BIOVIA) was first employed to create the molecular model of PVDF chain segments and MAPB nanocrystal nanocomposite in three-dimensional. The size of the simulation box is set as 145 Å × 135 Å × 200 Å, where a single MAPB nanocrystal with a radius of 50 Å locates in the center of the box and is wrapped by the PVDF chain segments. For MAPB crystal structure containing Frenkel defects, we moved a bromine atom in $PbBr_3$ tetrahedral into its interstitial. Next, the whole molecular structure is preliminarily optimized using the Forcite tool in MS, and all the atoms are given with information on atomic ID, molecular ID, charge, spatial position, topological relationship and so on. Then, we used msi2lmp tool to transfer the optimized molecular model to Large-scale Atomic/Molecular Massively Parallel Simulator (LAMMPS) package.

The potential for hybrid perovskites reported by Mattoni et al.[7] has been adopted here, which is the sum of organic-organic ($U_{\rm OO}$), inorganic-inorganic ($U_{\rm II}$) and organic-inorganic ($U_{\rm OI}$) interactions[8,9]. The $U_{\rm OO}$ term describes the intramolecular and intermolecular interactions of organic cations, which is based on the Assisted Model Building with Energy Refinement (AMBER) functional form and standard Generation Amber Force Field (GAFF) parameters. This force-field considers both the bonded and nonbonded interactions, where the bonded energy $U_{\rm bonded}$ describes the chemical group dissociation in molecular dynamics simulation and the non-bonded term $U_{\rm non\text{-}bonded}$ refers to the interaction between atoms that are not chemically bonded or separated by three or more bonds. The bonded interaction consists of three contributions and is expressed as:

$$\begin{aligned} U_{\rm bonded} &= U_{\rm bonds} + U_{\rm angles} + U_{\rm dihedrals} \\ &= \sum_{ij} \frac{1}{2} K_{ij}^{r} (r_{ij} - r_{ij}^{0}) + \sum_{ijk} \frac{1}{2} K_{ijk}^{\theta} (\theta_{ijk} - \theta_{ijk}^{0}) + \sum_{ijkl} \frac{1}{2} K_{ijkl}^{\phi} (1 + \cos(n_{ijkl}\phi_{ijkl} - \phi_{ijkl}^{0})) \end{aligned} \quad (7)$$

where subscripts *ijkl* represent atomic serial indices, and r_{ij}^{0}, θ_{ijk}^{0}, ϕ_{ijkl}^{0} represent the equilibrium values of bond length, bond angle and dihedral angle, respectively. K_{ij}^{r}, K_{ijk}^{θ}, K_{ijkl}^{ϕ} are force constants. The integer n_{ijkl} is the multiplicity associated to the torsion involving atoms *ijkl*. $U_{\rm non\text{-}bonded}$ includes Coulombic interactions and dispersive forces described by the 12-6 Lennard-Jones potential:

$$\begin{aligned} U_{\rm non\text{-}bonded} &= U_{\rm Coul} + U_{\rm vdW} \\ &= \sum_{ij} \frac{q_i q_j}{4\pi\varepsilon_0 r_{ij}} + \sum_{ij} 4\varepsilon_{ij} \left[\left(\frac{\sigma_{ij}}{r_{ij}} \right)^{12} - \left(\frac{\sigma_{ij}}{r_{ij}} \right)^{6} \right] \end{aligned} \quad (8)$$

where r_{ij} represents the distance between atom *i* with charge q_i and atom *j* with charge q_j. ε_0 is the vacuum dielectric constant, and ε_{ij} is the depth of the LJ potential well with σ_{ij}

the finite distance at which the inter-particle potential is zero. U_{II} term is used to describe ionic crystals, which consists of the Buckingham-Coulomb (BC) potential:

$$U_{\mathrm{II}} = \sum_{ij}\left[A_{ij}\exp\left(-\frac{r_{ij}}{\rho_{ij}}\right)-\frac{c_{ij}}{r_{ij}^{6}}\right]+\sum_{ij}\frac{q_i q_j}{4\pi\varepsilon_0 r_{ij}} \tag{9}$$

where ρ_{ij} is an ionic-pair dependent length parameter, which controls the repulsive term with prefactor A_{ij} and c_{ij} is the coefficient of attractive term. U_{IO} term is used to represent hybrid interactions, which can be described as the sum of three terms (Buckingham, Coulomb and Lennard-Jones 12-6) as follows:

$$U_{\mathrm{OI}} = \sum_{ij}\left[A_{ij}\exp\left(-\frac{r_{ij}}{\rho_{ij}}\right)-\frac{c_{ij}}{r_{ij}^{6}}\right]+\sum_{ij}\frac{q_i q_j}{4\pi\varepsilon_0 r_{ij}}+\sum_{ij}4\varepsilon_{ij}\left[\left(\frac{\sigma_{ij}}{r_{ij}}\right)^{12}-\left(\frac{\sigma_{ij}}{r_{ij}}\right)^{6}\right] \tag{10}$$

The intermolecular and intramolecular interactions of PVDF are described by the all-atom Consistent Valence Forcefield (CVFF)[10-12], which has been used successfully to simulate a wide variety of chemical and biophysical systems[13,14]. Here, bond stretching, bond angle bending and dihedral angle torsion potential energy are represented by harmonic potentials. The bond increment method is used to estimate the partial charge in CVFF[15], and the partial charge contributed from atom j to atom i is called bond increment Q_{ij}. Thus, the charge of atom i can be calculated by the sum of Q_{ij} as given in Eq. (11).

$$q_i = \sum_{j} Q_{ij} \tag{11}$$

where j runs over all the atoms which are bonded to atom i directly. The parameters of CVFF are assigned to the component of the simulated system using Forcite tool in MS. We use the geometric mixing rule ($\varepsilon_{ij} = \sqrt{\varepsilon_i\varepsilon_j}$, $\sigma_{ij} = \sqrt{\sigma_i\sigma_j}$) to express the interaction between PVDF and MAPB.

The molecular dynamics simulations were performed based the open source codes from LAMMPS and the data generated from msi2lmp. The velocity-Verlet integrator is used in the integration of the equations of motion using a time step of 0.5 fs. The Ewald method is used to describe the electrostatic interactions and the cut-off radius was set as 10 Å. Periodic boundary conditions are applied in three directions. The initial temperature of the system is set to 193.15 K. During the equilibration process, MAPB/PVDF nanocomposite system was relaxed from 193.15 K to 293.15 K for 100 ps in the canonical (NVT) ensemble. After that, the PVDF/MAPB system was further equilibrated at 293.15 K and 1.0 atm for another 100 ps in the constant-pressure, constant-temperature (NPT) ensemble, where the temperature was controlled by the Nosé–Hoover thermostat, and the pressure was controlled by the Nosé–Hoover barostat, till the total energy of the whole system reached a constant level. For the poling condition, an electric field of 400 V μm^{-1} along the y-axis was applied in situ during the relaxation process. For the observation on the interfaces between MAPB nanocrystals and PVDF chain segments, two PVDF chains closest to the surface of MAPB nanocrystals were selected.

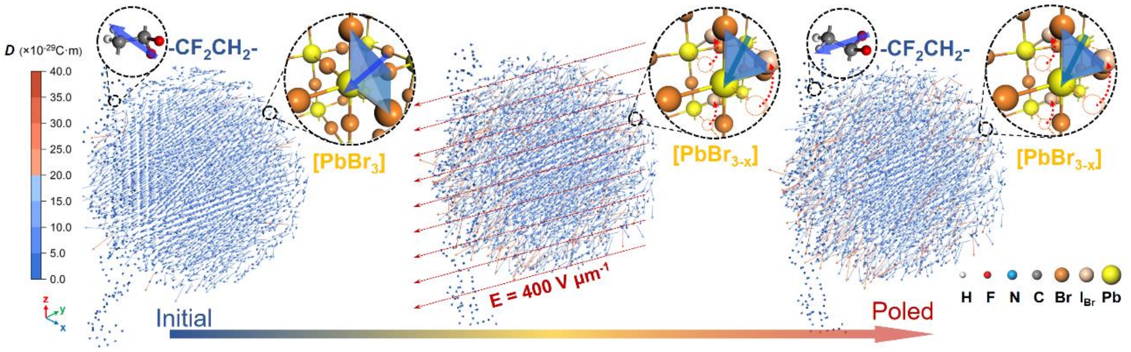

Supplementary Fig. 18 3D simulation on dipole evolution in MAPB/PVDF nanocomposite during poling. Dipoles aligned with poling electric field and Br Frenkel defects generated during poling causing large dipoles on MAPB nanocrystal surface,

stabilized by poled PVDF. The enlarged images showing the structural units of PVDF chain and MAPB crystal that generating C-F, [$PbBr_3$], [$PbBr_{3-x}$] dipoles, respectively.

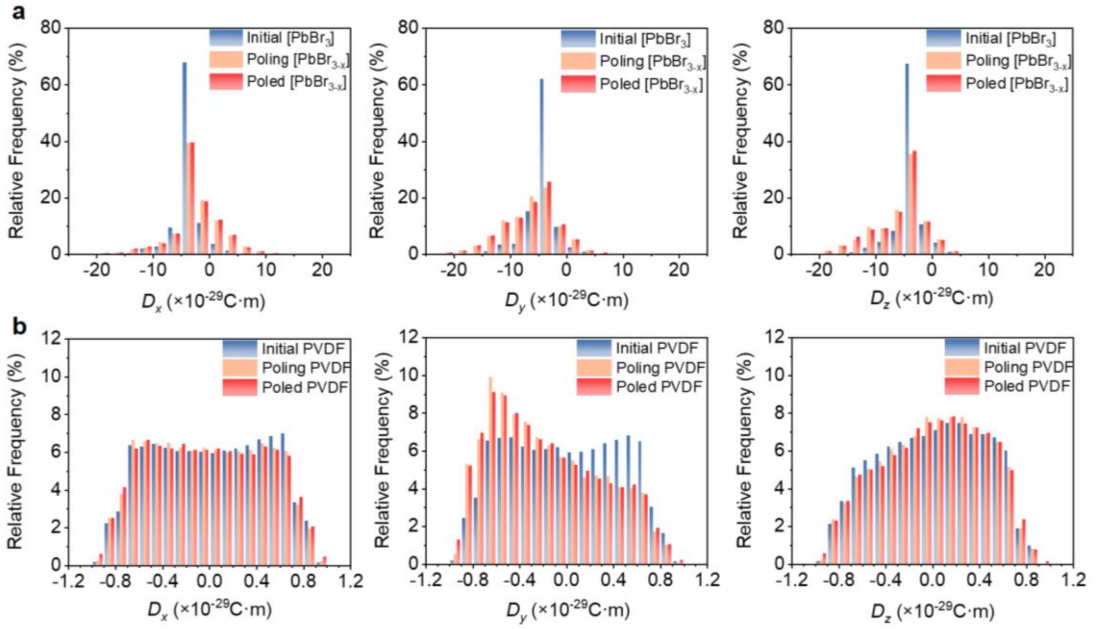

Supplementary Fig. 19 Comparison on the histograms of dipoles distribution of MAPB/PVDF nanocomposite with Br Frenkel defects generated during poling. **a** Components of dipole moment in three directions from initial [$PbBr_3$] and defected [$PbBr_{3-x}$] tetrahedron during poling and after poling, respectively. **b** Dipoles from CF_2 segment in PVDF along three directions. The poling electric field is applied along -y axis direction as shown in Fig. 5b.

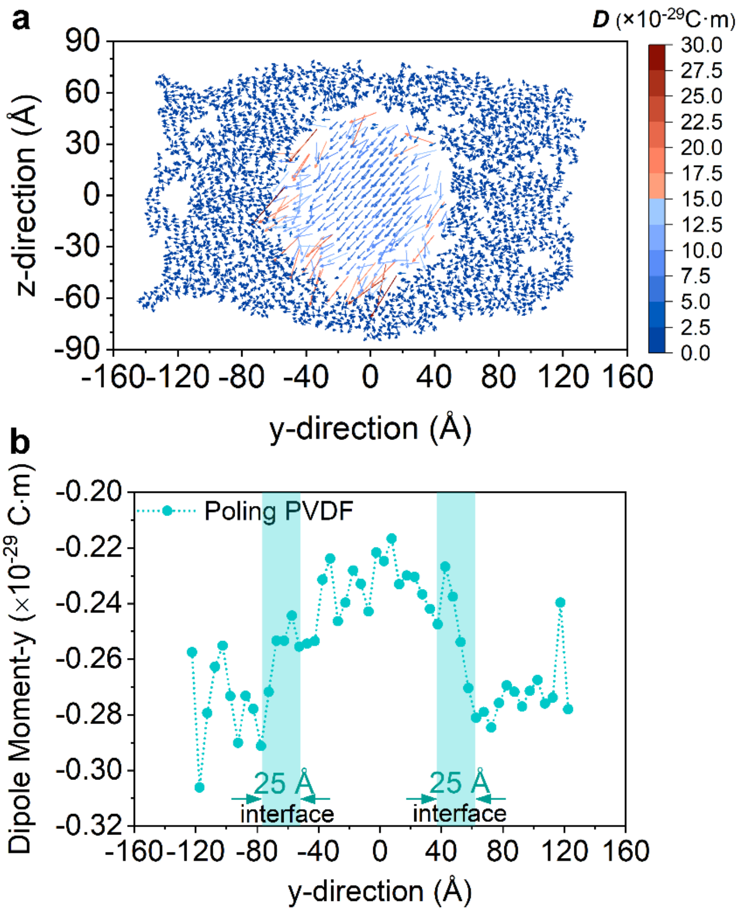

Supplementary Fig. 20 Observation of interfacial coupling in MAPB/PVDF from MD simulation in expanded volume of 300 Å × 270 Å × 200 Å under poling. a A slice of C-F and [$PbBr_3$] dipoles distribution in the yz plane. **b** Statistics on the sum of C-F dipole vectors from all the PVDF chains along y-axis direction.

Supplementary Note 13. Ferroelectric behavior of MAPB/P(VDF-HFP) nanocomposite film

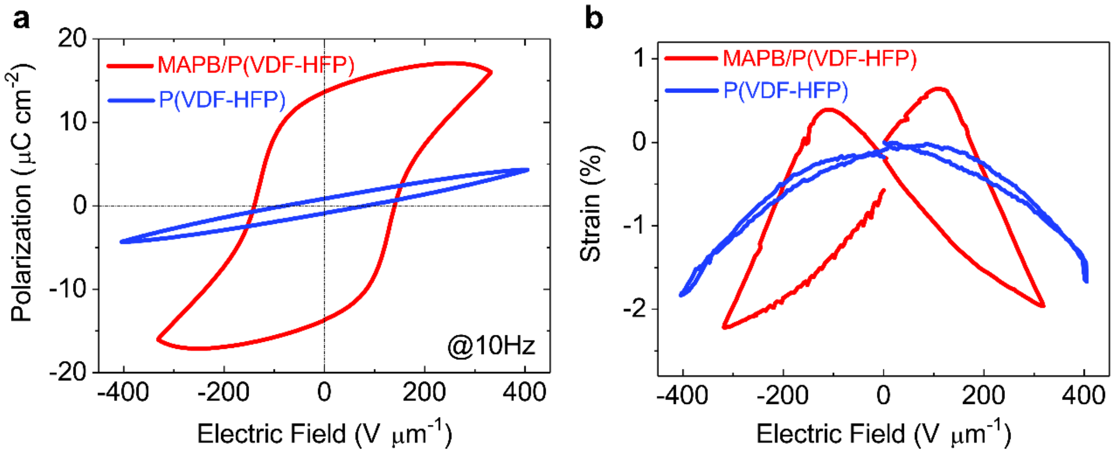

Supplementary Fig. 21 Electromechanical properties of MAPB/P(VDF-HFP) with 0.5% MAPB nanocrystals in comparison to P(VDF-HFP). **a** *P*-*E* curves. **b** Strain vs. electric field curves.

Supplementary References

1. Zhang, Y. et al. High-throughput scanning second-harmonic-generation microscopy for polar materials. *Adv. Mater.* **35**, 2300348 (2023).

2. Hasegawa, R., Takahashi, Y., Chatani, Y. & Tadokoro, H. Crystal structures of three crystalline forms of poly(vinylidene fluoride). *Polym. J.* **3**, 600-610 (1972).

3. Zhang, Y. et al. Characterization of domain distributions by second harmonic generation in ferroelectrics. *npj Comput. Mater.* **4**, 39 (2018).

4. Huang, B. et al. High-throughput sequential excitation for nanoscale mapping of electrochemical strain in granular ceria. *Nanoscale* **11**, 23188-23196 (2019).

5. Huang, B., Esfahani, E. N. & Li, J. Mapping intrinsic electromechanical responses at the nanoscale via sequential excitation scanning probe microscopy empowered by deep data. *Natl. Sci. Rev.* **6**, 55-63 (2019).

6. French, A. P. Vibrations and waves. New York: CRC Press, 1971.

7. Mattoni, A., Filippetti, A., Saba, M. I. & Delugas, P. Methylammonium rotational dynamics in lead halide perovskite by classical molecular dynamics: the role of temperature. *J. Phys. Chem. C* **119**, 17421-17428 (2015).

8. Mattoni, A., Filippetti, A. & Caddeo, C. Modeling hybrid perovskites by molecular dynamics. *J. Phys.: Condens. Matter* **29**, 043001 (2016).

9. Hata, T., Giorgi, G., Yamashita, K., Caddeo, C. & Mattoni, A. On the development of a classical interatomic potential for $MAPbBr_3$. *J. Phys. Chem. C* **121** (2017).

10. Plimpton, S. Fast parallel algorithms for short-range molecular dynamics. *J. Comput. Phys.* **117**, 1-19 (1995).

11.Ramos, M. M. D., Correia, H. M. G. & Lanceros-Méndez, S. Atomistic modelling of processes involved in poling of PVDF. *Comput. Mater. Sci.* **33**, 230-236 (2005).

12. Hu, T. The Predicted dielectric constant of an amorphous PVDF changing with temperature by molecular dynamics simulations. *Int. J. Electrochem. Sci.* **13**, 10088-10100 (2018).

13. Makowska-Janusik, M., Reis, H., Papadopoulos, M. G., Economou, I. G. & Zacharopoulos, N. Molecular dynamics simulations of electric field poled nonlinear

optical chromophores incorporated in a polymer matrix. *J. Phys. Chem. B* **108**, 588-596 (2004).

14. Hagler, A. T. & Lifson, S. Energy functions for peptides and proteins. II. Amide hydrogen bond and calculation of amide crystal properties. *J. Am. Chem. Soc.* **96**, 5327-5335 (1974).

15. Sun, H. Ab initio calculations and force field development for computer simulation of polysilanes. *Macromolecules* **28**, 701-712 (1995).
