# Supplementary material for: Halide Perovskite Inducing Anomalous Nonvolatile Polarization in Poly(vinylidene fluoride)-based Flexible Nanocomposites
Source: Nat Commun. 2024 May 10;15:3943. doi: 10.1038/s41467-024-48348-4 (PMC11087492; doi:10.1038/s41467-024-48348-4)
Supplement: Supplementary file 3 — Description of Additional Supplementary Files [file 41467_2024_48348_MOESM3_ESM.pdf]

Description of Additional Supplementary Files

Supplementary Movie 1: Nanocomposite film formation.
